# Attitude towards working in rural area and self-assessment of competencies in last year medical students: A survey of five countries in Asia

**DOI:** 10.1186/s12909-016-0719-9

**Published:** 2016-09-07

**Authors:** Wanicha L. Chuenkongkaew, Himanshu Negandhi, Pisake Lumbiganon, Weimin Wang, Kawkab Mahmud, Pham Viet Cuong

**Affiliations:** 1 Department of Ophthalmology, Faculty of Medicine, Siriraj Hospital, Mahidol University, Bangkok, Thailand 10700; 2 Public Health Foundation of India, Delhi NCR, Plot No. 47, Sector 44, Institutional Area, Gurgaon, 122002 India; 3 Department of Obstetrics and Gynecology, Faculty of Medicine, Khon Kaen University, Khon Kaen, Thailand 40002; 4 Peking University Health Science Center, Beijing, China; 5 James P Grant School of Public Health, BRAC University, Dhaka, Bangladesh; 6 Hanoi School of Public Health, Hanoi, Vietnam

**Keywords:** Medical student, Attitude, Competencies, Rural areas, Asian countries

## Abstract

**Background:**

Five countries in Asia including Bangladesh, China, India, Thailand and Vietnam formed a network called Asia-Pacific Network for Health Professional Education Reforms (ANHER). This network collectively conducted a survey at the national level and at the institutional level (for medical, nursing and public health education). We also undertook an assessment of final year graduates from these schools on their attitudes, competencies and willingness to work in rural areas.

**Methods:**

Pretested anonymous questionnaire comprised of four sections including demographic data, attitudes towards working in rural area, where to work after graduation and perception about competency of respondents was used. Descriptive and analytical statistics were used for data analyses.

**Results:**

About 60 % of students from Bangladesh and Thailand had positive attitude towards working in rural area, 50 % in both China and India and only 33 % in Vietnam. Students’ positive attitudes towards their school in terms of preparing or inspiring them to work in rural areas were low across all five countries. Upon graduation and in the next five years, majority of students wanted to work in public sectors. Interestingly confidence about overall competency was quite low.

**Discussion:**

Positive attitude towards working in rural areas varied significantly across five countries in Asia. Medical schools should improve the preparation and inspiration towards working in rural areas for their students.

**Conclusion:**

Medical schools should put more effort in improving students’ attitude towards working in rural areas.

**Electronic supplementary material:**

The online version of this article (doi:10.1186/s12909-016-0719-9) contains supplementary material, which is available to authorized users.

## Background

*The Lancet Commission on Health Professionals for the 21st Century* was launched on the 100th anniversary of the Flexner Report which proposed a replacement of a science based curriculum by a system-based approach in medical education.[1] This prompted a solid movement in health education reform in different countries, including five Asian countries, namely Bangladesh, China, India, Thailand and Vietnam which are located in the World Health Organization (WHO) South East Asia Region (SEAR) and Western Pacific Region (WPR). These five countries formed a network called Asia-Pacific Network for Health Professional Education Reforms (ANHER) to reform health professional education. The members collectively developed and adopted a common protocol and tools to survey their national scenario and institutional levels of medical, nursing and public health education; assess the attitudes, competencies and willingness among their final year graduates to serve rural health services.. These evidences enabled a platform for national strategies to transform their health professional education systems to be more responsive to population health needs. The global emerging challenges include demographic and epidemiologic transitions, technology-driven health care systems unwarranted fragmentation and overly sub-specialization of care, cultural situation as well as domestic health systems dynamics. In 2010, the WHO addressed that approximately one-half of the world population lived in rural area while less than a quarter of physicians worked in remote and rural area despite adequate absolute number of physicians in some low-income countries.[2] The proportion of rural population of Bangladesh, China, India, Thailand and Vietnam in 2014 were 66, 68, 51, 67 and 46 % respectively. [3] The WHO recommended 22.8 as the density threshold of physicians, nursing and midwifery personnels per 10000 population. [4] This indicator in 2014 for Bangladesh, China, India, Thailand and Vietnam were 5.8, 29.7, 24.1, 24.7 and 23.0 respectively. [5] At the time of this survey there were no expected proportion of students in Bangladesh, China and India willing to take up rural practice. All medical graduates in Thailand were expected to serve public work mainly in rural areas for three year. In Vietnam, only 1.5 % of medical graduates were expected to work for the government in rural areas. A more complicated problem is about mal-distributed health cadres and domestic as well as international migration.[6] A recent survey of Thai medical, dental and pharmacy new graduates in 2012 showed that the overall attitudes towards working in rural areas were quite positive. [7] Medical students in the capital city of Ethiopia had poor attitude towards working in rural areas and majority of them intended to migrate to work abroad mainly in the US and Europe. [8] A cross sectional survey was conducted among final-year medical students at premier government institutions in South Asia (Bangladesh, India, Nepal) and Sub-Saharan Africa (Ethiopia, Kenya, Malawi, the United Republic of Tanzania and Zambia). The overall intention to pursue a rural career was only 15 % (17 % in South Asia and 13 % in Sub-Saharan Africa). [9] A survey conducted among 169 medical students enrolled in Rural Clinical School Program at University of New South Wales in Australia indicated that only about 30 % of these medical students intended to work in rural or remote areas. [10] Cross sectional survey conducted in 792 first year medical students in Madhya Pradesh Province of India revealed that 91 % of students wished to pursue a specialization and 64 % planned to work in urban areas. [11] Our study was aimed at evaluating medical students’ attitudes towards working in rural and remote areas, attitudes towards their school in terms of preparing or inspiring them to work in rural areas, type of work they want to do upon graduation and the next five years and their confidence in overall competency. We also wanted to explore factors associated with these issues.

## Methods

### Study design

#### Descriptive and cross sectional analytical study

##### Population

Last year medical students just before graduation in the year 2012 from medical schools in Bangladesh, China, India, Thailand and Vietnam.

##### Sampling

In Bangladesh, classroom census of all medical students studying in the final year (5th year) of their undergraduate course from 23 medical colleges. These medical colleges were selected purposively considering the proportion of public and private medical colleges from all over the country. In China, stratified random sampling by region and types of institution was conducted to obtain 10 % of all institutions. Furthermore, another three leading institutions were purposely selected for the survey as being suggested by two experts in medical education after they reviewed the sampling cohort. In India, we performed a stratified random sampling by geographical region to obtain 10 medical colleges from each of the four regions. In Thailand it was a census of medical students of all 19 medical schools in 2012. In Vietnam sample size was calculated based on the single proportion estimates and was adjusted to ensure representation and geographical distribution area. Sampling for institution was randomly selected from sampling allocation table. Subjects were selected randomly based on student lists.

##### Data collection

We organized a workshop involving researchers from all five countries in Khon Kaen, Thailand to develop the study questionnaire. We pre-tested the questionnaire in a group of medical students in all five countries and modified the questionnaire accordingly. Countries had the flexibility to carry out minor adaptations in the questionnaires as per the country-situation. The survey questionnaire comprised of four sections. The first section was about demographic data including sex, age, and residential location, occupation of father and mother and mode of admission to medical school. The second part was related to attitudes towards working in rural areas and the extent that their medical school had prepared them to work in rural areas using one to five Likert scale. The answers will be grouped into two categories, ‘positive’ for any answer with the scale 4 or 5, and ‘not positive’ if otherwise. The third section asked respondents about where to work upon graduation and in the next five years. The last section was self-assessment of competencies covering administrative competencies, interprofessional skill, communication skill overall clinical skill and overall public health skill. All items were arranged to 1 (not confident at all to 5 (very confident) Likert scale. Mean score at 4 or above will be considered ‘confident’, while score below 4 will be justified as ‘not confident’. The pretested questionnaires were distributed to all randomly selected participants. The questionnaire was anonymous and measured respondent’s attitudes subjectively.

### Data analysis

The analysis procedure constitutes three parts. Firstly, descriptive statistics were used on demographic data. Demographic information was described in ‘percentage’ and ‘mean’. Secondly, univariate analyses were performed by cross tabulation between each demographic attribute with each dependent variable (rural attitude in section 2, intention to work in section 3, and competency in section 4). Multivariate analysis was conducted using independent variables which yielded statistically-significant results in the descriptive analysis were included in a multilevel fixed effect logistic regression. Country was deemed as cluster (higher level variable). Therefore, the association was already adjusted for cluster effect.

## Results

### Descriptive statistics

There were altogether 7945 respondents, 1422, 3045, 1978, 1238 and 262 from Bangladesh, China, India, Thailand and Vietnam respectively. The proportions of female students were more than male in all countries except India ranging from 60 % in China to 48 % in India. Mean age ranged from 22.7 years in Bangladesh to 24.9 years in Thailand. A majority of the students in Bangladesh, India and Thailand spent their childhood period in urban areas, while in China and Vietnam, most respondents were in rural areas, Table 1. During high school period most students in all countries lived in urban or suburban areas, Table 1. Majority of parents in Bangladesh, India and Thailand lived in urban areas while those of China and Vietnam were in rural areas, Table 1. Fathers of majority of students in China and Vietnam were non-professional, 70 % of the fathers in Bangladesh were professional, while it was quite balanced in India and Thailand, Table 2. 70 to 80 % of students’ mothers in Bangladesh, China, India and Vietnam were non-professional; the corresponding figure in Thailand was almost 60 %, Table 2. Concerning fathers’ education, majority (67 to 79 %) of students’ fathers from Bangladesh, India and Thailand completed bachelor degree or higher, the figures in China and Vietnam were 31 and 47 % respectively, Table 2. In general, mothers’ education was lower than fathers’ education except in Thailand where their levels of educations were similar, Table 2. 61.2, 95.6, 70.2, 36.0 and 85.9 % of students in Bangladesh, China, India, Thailand and Vietnam respectively were admitted through national entrance examination.

**Table 1:** Residence during childhood and high school and residence of parents

Country	Residence during childhood			Residence during high school			Residence of parents		
	Rural No (%)	Semi-urban No (%)	Urban No (%)	Rural No (%)	Semi-urban No (%)	Urban No (%)	Rural No (%)	Semi-urban No (%)	Urban No (%)
Bangladesh	219 (15.8)	184 (13.3)	981 (70.9)	143 (10.3)	211 (15.2)	1,033 (74.5)	201 (14.4)	151 (10.9)	1,041 (74.7)
China	1,485 (50)	753 (25.3)	733 (24.7)	313 (10.5)	1,756 (59.1)	901 (30.4)	1,297 (43.9)	813 (27.5)	845 (28.6)
India	316 (16.0)	553 (27.9)	1,109 (56.1)	170 (8.6)	537 (27.1)	1,271 (64.3)	277 (14.0)	403 (20.4)	1,298 (65.6)
Thailand	228 (20.3)	183 (16.3)	713 (63.4)	53 (4.7)	110 (9.8)	959 (85.5)	236 (19.7)	214 (17.9)	746 (62.4)
Vietnam	137 (52.3)	54 (20.6)	71 (27.1)	66 (25.3)	95 (36.4)	100 (38.3)	129 (49.2)	48 (18.3)	85 (32.5)
Total	2,385 (30.9)	1,727 (22.4)	3,607 (46.7)	745 (9.7)	2,709 (35.1)	4,264 (55.2)	2,140 (27.5)	1,629 (20.9)	4,015 (51.6)

**Table 2:** Fathers’ and Mothers’ occupation and education

Country	Occupation				Education			
	Father		Mother		Father		Mother	
	P No (%)	NP No (%)	P No (%)	NP No (%)	>Bachelor degree No (%)	<Bachelor degree No (%)	>Bachelor degree No (%)	<Bachelor degree No (%)
Bangladesh	1,000 (70.3)	422 (29.7)	340 (23.9)	1,082 (76.1)	1062 (74.8)	357 (25.2)	662 (46.8)	753 (53.2)
China	666 (22.7)	2,268 (77.3)	578 (19.8)	2,336 (70.2)	936 (31.2)	2,059 (68.8)	694 (23.4)	2274 (76.6)
India	1,015 (51.3)	963 (48.7)	425 (21.5)	1,553 (78.5)	1,554 (78.6)	424 (21.4)	1,179 (59.7)	798 (40.3)
Thailand	556 (48.6)	587 (51.4)	492 (42.8)	658 (57.2)	795 (67.0)	392 (33.0)	773 (64.3)	430 (35.7)
Vietnam	101 (38.6)	161 (61.4)	77 (29.7)	182 (70.3)	116 (46.8)	132 (52.2)	93 (36.9)	159 (63.1)
Total	3,338 (43.1)	4,401 (56.9)	1,912 (24.8)	5,811 (75.2)	4,463 (57.0)	3,364 (43.0)	3,402 (43.5)	4,414 (56.5)

*P* Professional, *NP* Non Professional

About 60 % of students from Bangladesh and Thailand had positive attitude towards working in rural area. The corresponding figures were 50 % in both China and India. Interestingly only 33 % of students in Vietnam had positive attitudes, Table 3. Students’ positive attitudes towards their school in terms of preparing or inspiring them to work in rural areas were quite low across all five countries, ranging from 22 % in Vietnam to 46 % in China, Table 3. Interestingly the positive attitude about overall competency was quite low ranging from 25 % in India to 43 % in Bangladesh, Table 3.

**Table 3:** Attitude towards working in rural areas, attitude towards school in preparing or inspiring to work in rural areas and attitude about overall competency

Country	Attitude					
	towards working in rural areas		towards school in preparing or inspiring to work in rural areas		about overall competency	
	Positive No (%)	Not Positive No (%)	Positive No (%)	Not Positive No (%)	Positive No (%)	Not Positive No (%)
Bangladesh	984 (69.2)	438 (30.8)	647 (45.5)	775 (54.5)	607 (42.7)	815 (57.3)
China	1,425 (46.8)	1,620 (53.2)	1,010 (33.2)	2,035 (66.8)	1,288 (42.3)	1,757 (57.7)
India	1,020 (51.6)	958 (48.4)	640 (32.4)	1,338 (67.6)	505 (25.5)	1,473 (74.5)
Thailand	856 (69.1)	382 (30.9)	530 (42.8)	708 (57.2)	327 (26.4)	911 (73.6)
Vietnam	86 (32.8)	176 (67.2)	58 (22.1)	204 (77.9)	82 (31.3)	180 (68.7)
Total	4,371 (55.0)	3,574 (45)	2,885 (36.3)	5,060 (63.7)	2,809 (35.4)	5,136 (64.6)

Regarding immediate job upon graduation, majority of students wanted to work in public sectors ranging from 57 % in China to 92 % in Thailand, Table 4. Pro-public job preference in the next five years range from 60 % in India to 86 % in Thailand, Table 4.

**Table 4:** Upon graduation and next-five year job preference

Country	Job preference			
	Upon graduation		Next-five year	
	Pro Public Sector No (%)	Not Pro Public Sector No (%)	Pro Public Sector No (%)	Not Pro Public Sector No (%)
Bangladesh	944 (71.9)	369 (28.1)	872 (65.6)	457 (34.4)
China	1,678 (57.1)	1,262 (42.9)	2,184 (73.6)	783 (26.4)
India	1,243 (62.8)	735 (37.2)	1,095 (60.0)	731 (40.0)
Thailand	1,098 (92.4)	90 (7.6)	1,029 (85.5)	174 (14.5)
Vietnam	231 (88.2)	31 (11.8 %)	216 (83.1)	44 (16.9)
Total	5,194 (67.6)	2,487 (32.4)	5,396 (71.1)	2,189 (28.9)

### Inferential statistics

We observed the following by undertaking univariate analyses. Factors associated with attitude towards working in rural area include gender, residence during childhood and high school, parents’ residence, professions of father and mother, father’s and mother’s education and mode of admission into medical schools, Table 5. Factors associated with positive attitudes towards their school in terms of preparing or inspiring them to work in rural areas includes parents’ residence and mode of admission into medical schools, Table 5. Factors associated with intention to work in public sector upon graduation includes age, residence during childhood and high school, parents’ residence, professions of father and mother, father’s and mother’s education and mode of admission into medical schools, Table 6. Factors associated with intention to work in public sector five years after graduation include age, residence during childhood, parents’ residence, professions of father and mother, father’s education and mode of admission into medical schools, Table 6. Factors associated with attitude about overall competency include gender, residence during childhood and high school, parents’ residence, professions of father, fathers’ and mothers’ education and mode of admission into medical schools, Table 7.

**Table 5:** Factors associated with attitude towards working in rural areas and attitude towards school in preparing for working in rural areas (Univariate analyses)

Factors		Positive attitude towards working in rural areas (%)	*P*-value	Positive attitude towards school in preparing for working in rural areas (%)	*P*-value
Gender	Female	2459/4432 (55.5 %)	0.000	1590/4342 (36.6 %)	0.252
	Male	1864/3539 (52.7 %)		1252/3539 (35.4 %)	
Residence during childhood	Rural	1189/2385 (49.9 %)	0.000	833/2385 (34.9 %)	0.148
	Semi-urban	923/1727 (53.4 %)		601/1727 (34.8 %)	
	Urban	2118/3607 (58.7 %)		1335/3607 (37.0)	
Residence during high school	Rural	390/745 (52.3 %)	0.000	284/745 (38.1 %)	0.128
	Semi-urban	1,304/2709 (48.1 %)		934/2709 (34.5 %)	
	Urban	2536/4264 (59.5 %)		1544/4264 (36.2 %)	
Parents’ residence	Rural	1052/2140 (49.2 %)	0.000	729/2140 (34.1 %)	0.018
	Semi-urban	897/1629 (55.1 %)		628/1629 (38.6 %)	
	Urban	2326/4015 (57.9 %)		1444/4015 (36.0 %)	
Father’s occupation	Non Professional	2298/4401 (52.2 %)	0.000	1550/4401 (35.2 %)	0.119
	Professional	1942/3338 (58.2 %)		1233/3338 (36.9 %)	
Mother’s occupation	Non Professional	3126/5811 (53.8 %)	0.000	2066/5811 (35.6 %)	0.114
	Professional	1109/1912 (58.0 %)		718/1912 (37.6 %)	
Father’s education	< bachelor degree	1726/3364 (51.3 %)	0.000	1202/3364 (35.7 %)	0.678
	≥ bachelor degree	2570/4463 (57.6 %)		1615/4463 (36.2 %)	
Mother’s education	< bachelor degree	2347/4414 (53.2 %)	0.001	1563/4414 (35.4 %)	0.256
	≥ bachelor degree	1942/3402 (57.1 %)		1247/3402 (36.7 %)	
National entrance	No	1324/2104 (62.9 %)	0.000	948/2104 (45.1 %)	0.000
	Yes	2015/4796 (42.0 %)		1909/5796 (32.9 %)

**Table 6:** Factors associated with intention to work in public sector immediately and five years after graduation (univariate analyses)

Factors		Pro-public at immediately after graduation (%)	*P*-value	Pro-public at 5 years after graduation (%)	*P*-value
Gender	Female	2831/4234 (66.9 %)	0.111	2998/4168 (71.9 %)	0.089
	Male	2342/3415 (68.6 %)		2373/3383 (70.1 %)	
Residence during childhood	Rural	1472/2320 (63.4 %)	0.000	1705/2307 (73.9 %)	0.003
	Semi-urban	1158/1688 (68.6 %)		1180/1668 (70.7 %)	
	Urban	2446/3493 (70.0 %)		2391/3427 (69.8 %)	
Residence during high school	Rural	496/740 (67.0 %)	0.138	496/729 (68.0 %)	0.147
	Semi-urban	1650/2647 (62.3 %)		1900/2624 (72.4 %)	
	Urban	2929/4133 (70.9 %)		2873/4055 (70.9 %)	
Parents’ residence	Rural	1354/2085 (64.9 %)	0.001	1530/2081 (73.5 %)	0.203
	Semi-urban	1086/1596 (68.0 %)		1132/1579 (71.7 %)	
	Urban	2701/3881 (69.6 %)		2667/3812 (70.0 %)	
Father’s occupation	Non Professional	2824/4285 (65.9 %)	0.000	3089/4270 (72.3 %)	0.008
	Professional	2266/3235 (70.0 %)		2197/3161 (69.5 %)	
Maternal occupation	Non Professional	3752/5646 (66.5 %)	0.000	3911/5583 (70.1 %)	0.001
	Professional	1326/1861 (71.3 %)		1358/1828 (74.3 %)	
Father’s education	< bachelor	2146/3274 (65.5 %)	0.001	2409/3276 (73.5 %)	0.000
	≥ bachelor	2996/4337 (69.1 %)		1939/3237 (59.9 %)	
Maternal education	< bachelor	2838/4295 (66.1 %)	0.086	3067/4264 (71.9 %)	0.094
	≥ bachelor	2308/3307 (69.8 %)		2271/3237 (70.2 %)	
National entrance	No	1453/1985 (73.2 %)	0.000	1327/1969 (67.4 %)	0.000
	Yes	3726/5671 (65.7 %)		4051/5589 (72.5 %)

**Table 7:** Factors associated with attitude about confidence in overall competency (univariate analyses)

Factors		Confidence (%)	*P*-value
Gender	Female	1388/4342 (32.0 %)	0.000
	Male	1376/3539 (38.9 %)	
Residence during childhood	Rural	894/2385 (37.5 %)	0.003
	Semi-urban	614/1727 (35.6 %)	
	Urban	1199/3607 (33.2 %)	
Residence during high school	Rural	279/745 (37.4 %)	0.000
	Semi-urban	1025/2709 (37.8 %)	
	Urban	1403/4264 (32.9 %)	
Parents’ residence	Rural	807/2140 (37.7 %)	0.000
	Semi-urban	605/1629 (37.1 %)	
	Urban	1320/4015 (32.9 %)	
Father’s occupation	Non Professional	1608/4401 (36.5 %)	0.003
	Professional	1112/3338 (33.3 %)	
Maternal occupation	Non Professional	2057/5811 (35.4 %)	0.343
	Professional	654/1912 (34.2 %)	
Father’s education	< bachelor	1281/3364 (38.1 %)	0.000
	≥ bachelor	1468/4463 (32.9 %)	
Maternal education	< bachelor	1620/4414 (36.7 %)	0.001
	≥ bachelor	1122/3402 (33.0 %)	
National entrance	No	650/2104 (30.9 %)	0.000
	Yes	2127/5796 (36.7 %)	

After adjusted for potential confounding factors and cluster effect using multilevel fixed effect logistic regression the following findings were observed. Factors significantly associated with attitude towards working in rural area include gender, residence during high school and parents’ residence. Female students were more likely to have positive attitude than male students. Students who lived in semi-urban areas during their high school period had significantly lower positive attitude compared to those living in rural areas. Students whose parents lived in semi-urban areas had significantly higher positive attitude compared to those living in rural areas. The only factor significantly associated with positive attitudes towards their school in terms of preparing or inspiring them to work in rural areas is parents’ residence. Students with parents living in semi-urban areas had significantly higher positive attitude compared to those living in rural areas. Factors significantly associated with intention to work in public sector upon graduation include residence during childhood and high school and mode of admission into medical schools. Students living in semi-urban areas during their childhood period had significantly more intention to work for public sector while students living in semi-urban areas during high school had less intention to work for public sector. Students admitted by national entrance examination had significantly more intention to work for public sector. Factors significantly associated with intention to work in public sector five years after graduation includes residence during childhood and mode of admission into medical schools. Students living in urban areas during their childhood period had significantly less intention to work for public sector while students admitted by national entrance examination had significantly more intention to work for public sector. Factors significantly associated with confidence in overall competency include gender and mother’s education. Male students and students whose mothers had bachelor degree or higher had significantly higher confidence in overall competency compared to female students and students whose mothers had less than bachelor degree respectively, Table 8.

**Table 8:** Factors associated with attitude towards working in rural areas, attitude towards school in preparing or inspiring to work in rural areas and attitude, job preference immediately and five years after graduation and overall competency (multivariate analyses)

Attitudes	Factors	Odds ratio (95 % CI)
1. Working in rural areas	Male gender	0.88 (0.86, 0.98)
	High school residence in semi-urban area	0.83 (0.69, 0.99)
	Parents’ residence in semi-urban area	1.34 (1.10, 1.62)
2. School in preparing to work in rural area	Parents’ residence in semi-urban area	1.18 (1.03, 1.35)
3. Job preference immediately after graduation	Childhood residence in semi-urban area	1.34 (1.10, 1.64)
	High school residence in semi-urban area	0.78 (0.64, 0.95)
	National entrance	1.30 (1.13, 1.49)
4. Job preference five years after graduation	Childhood residence in urban area	0.81 (0.66, 0.99)
	National entrance	1.57 (1.37, 1.80)
5. Overall competency	Male gender	1.39 (1.26, 1.54)
	Mother education (Bachelor degree or higher)	1.19 (1.05, 1.36)

## Discussion

The proportions of female students were more than male in all countries except India. Mean age ranged from 22.7 years in Bangladesh to 24.9 years in Thailand. About 60 % of students from Bangladesh and Thailand had positive attitude towards working in rural area, 50 % in both China and India and only 33 % in Vietnam. Students’ positive attitudes towards their school in terms of preparing or inspiring them to work in rural areas were quite low across all five countries. Upon graduation and in the next five years majority of students wanted to work in public sectors. Interestingly confidence about overall competency was quite low.

Factors significantly associated with attitude towards working in rural area include gender, residence during high school and parents’ residence. The only factor significantly associated with positive attitudes towards their school in terms of preparing or inspiring them to work in rural areas is parents’ residence. Factors significantly associated with intention to work in public sector include residence during childhood and high school and mode of admission into medical schools. Factors significantly associated with confidence in overall competency include gender and mother’s education.

Regarding positive attitude towards working in rural or remote area our study revealed a wide variation across five countries. The main reason might be because of very much difference in context between countries. However, majority of students wanted to work in public sectors both upon graduation and five years later. A report from Ethiopia showed that attitude of medical students towards working in rural areas were very poor and the intention to internationally migrate was very high. Only 30 % of these students would like to start practicing medicine in rural areas. [8] A recent survey of 3199 medical and nursing students from Bangladesh, Ethiopia, India, Kenya, Malawi, Nepal, the United Republic of Tanzania and Zambia indicated that only 16 % of these students plan to work in rural areas. [9]

Our study also found that students’ positive attitudes towards their school in terms of preparing or inspiring them to work in rural areas as well as confidence about overall competency were quite low across all five countries. Responsible authorities of these medical schools should be informed about this very important issue. In 2010, WHO published a global policy recommendation on “Increasing access to health workers in remote and rural areas through improve retention”. Education recommendations included 1) enroll students with rural background, 2) locate health professional schools, campuses and family medicine residency program outside of capitals and other major cities, 3) expose undergraduate students to rural community experience and clinical rotation, 4) revise under and postgraduate curricula to include rural health topics and 5) design continuing education and professional development program that meet the needs of rural health workers and that are accessible from where they live and work. [2] Challenges and lesson learnt in implementing this recommendation in Lao People Democratic Republic and South Africa was reported recently. [12] An online survey of 156 last year medical students in the United States concluded that students had performed most basic skills infrequently and had no confidence in performing these skills without assistance, similar to our observation.[13] In addition, self-assessment of clinical competency by graduates in Yemen was found to be overestimated comparing to expert’s assessment. [14] Problem-based learning at the institute has an impact upon graduate competency. [15]

Factors associated with attitude towards working in rural area included gender, domicile during high school and parents’ domicile and those with intention to work in public sector included domicile during childhood and high school and mode of admission into medical schools. A retrospective cohort study from Nepal reviewed that factors associated working in rural area included paramedical background, rural domicile at birth and poor academic performance. [16] The recent study in India did not find significant association between graduation from public or private medical schools and job preference in the future.[11] In Ethiopia, students with rural background were more likely to intend to work in rural areas compared to those with urban background. [8]

### Limitations of the study

This is a multi-country studies involving five major countries in Asia which represent more than one half of world population. Although common protocols were used, contribution varied in different countries from 3.3 to 38.3 %. Furthermore, rural, urbanized and semi-urbanized areas were self-defined country by country which might not be exactly the same. However, this issue is very much context specific and investigators in the country would have clear ideas what rural, sub-urban and urban means in their context. All five countries defined rural area as area that is located outside towns and cities.

## Conclusion

Medical students’ low positive attitudes towards their school in inspiring them to work in rural area as well as their low confidence in overall competency to work in rural area should strongly alert administrative authorities of medical schools. Appropriate strategies including more emphasis on community and competency-based learning should be implemented based on local context. Medical schools should provide more learning opportunities to improve students’ confidence by implementing adequate simulation and early clinical exposure in learning processes e.g. rotation to community hospitals for clinical skills and more engagement in health system.

## Additional file

Additional file 1: Student survey. (PDF 132 kb)

## Abbreviations

ANHER: Asia-Pacific Network for Health Professional Education Reforms (ANHER)
SEAR: South East Asia Region
WHO: World Health Organization
WPR: Western Pacific Region
