# Supplementary material for: Attitude towards working in rural area and self-assessment of competencies in last year medical students: A survey of five countries in Asia
Source: BMC Med Educ. 2016 Sep 7;16(1):238. doi: 10.1186/s12909-016-0719-9 (PMC5015323; doi:10.1186/s12909-016-0719-9)
Supplement: Additional file 1: — Student survey. (PDF 132 kb) [file 12909_2016_719_MOESM1_ESM.pdf]

Tool 3a Survey of medical students who are about to graduate

December 11, 2011

Objectives

1. To assess the relationship between socio-economic background, attitudes towards working in rural and remote areas, and types of health service employment after graduate [public, private, service, teaching, administration] .
2. To conduct a self assessment by potential graduate on knowledge, skill and competencies and their experiences during the medical education course.

Methods

It is easy to identify the last year medical students who are about to finish the course. A self-administrative questionnaire survey can be applied in class with almost 100% responses rate. This sample provides good information about the young graduates and found useful for policy reorientation by the medical schools and policy makers. There is an opportunity to assess the curriculum and training modality as they have fresh experiences.

Tool 3a Medial students who are about to leave school

A self-administered survey questionnaire is launched to all graduates who are about to leave the school. Tool should be tested, verify its reliability, and validity for a final version. A class room type of self-administration questionnaire survey can be applied easily, without intervention by surveyors. Collection of questionnaire at the end of the class would ensure almost 100% response rate.

Key questions

Questions would be adjusted according to the country context, while keep several core questions for cross country comparisons.

To answer the following questions, please indicate ☒ in box or write down your reply in the appropriate space.

1. About yourself

1.1 Gender ☐ 1. Male ☐ 2. Female

1.2 Year of birth [............]

1.3 Please indicate your current study program

☐1. MBBS program

☐2. MD program

1.4When you were 1-15 years, where did you spend most of your life? [Please modify answers according to country contexts]

☐1. Village

☐2. Small town

☐3. City

☐4. Other, specify........................

1.5 Where was your high school located? [Please modify answers according to country contexts]

☐1. Village

☐2. Small town

☐3. City

☐4. Country capital

☐5. Other, specify........................

1.6 You were recruited into medical school through what mechanism [Please design according to your country context]

☐1. National entrance exam

☐2. Direct admission through a specific institution

☐3. Special quota

☐4. Others, specify........................

2. About your parents

2.1 Where do they live now? [Please modify answers according to country contexts]

❑1. Village

❑2. Small town

❑3. City

❑4. Other, specify

2.2 Main occupation of your father and mother[1] [Research team may use country specific nomenclature of occupation and then re-classified according to ISCO 2 digits level of detail later e.g. choice 1 health professionals = 22, choice 2 agricultural labourers = 92]

2.2a Occupation of your father	2.2b Occupation of your mother
❑1. XXX	❑1. XXX
❑2. XXX	❑2. XXX
❑3. XXX	❑3. XXX
❑4 XXX	❑4. XXX
❑5. XXX	❑5. XXX

[1] Adapted from the classification by International Standard Classification of Occupations (ISCO) http://www.ilo.org/public/english/bureau/stat/isco/docs/resol08.pdf

2.3 What are the highest education level of your father and mother[2]?

**2.3a Highest education level of your father**	**2.3b Highest education level of your mother**
❑1. Never attended school	❑1. Never attended school
❑2. Primary school	❑2. Primary school
❑3. Secondary school	❑3. Secondary school
❑4. Diploma/Vocational level	❑4. Diploma/Vocational level
❑5. Bachelor level	❑5. Bachelor level
❑6. Master level	❑6. Master level
❑7. Doctoral level	❑7. Doctoral level
❑8. Others, specify………..	❑8. Others, specify………..

[2] Adapted from the classification by The International Standard Classification of Education (ISCED) http://www.uis.unesco.org/Education/Documents/UNESCO_GC_36C-19_ISCED_EN.pdf

3. Perception/Attitudes towards rural, remote or hardship areas

**Please read these statements very carefully and decide whether you agree or disagree. Note that "these areas" refers to work in rural, remote or hardship areas as defined by individual country**	**←Disagree -----------Agree→**				
	1	2	3	4	5
1. Working in these areas provides opportunities to use various skills					
2. There are supportive environment when working in these areas					
3. Working in these areas limits communications with professional peers					
4. Working in these areas provides opportunities to work independently					
5. There are lack of amenities and entertainment in these areas					
6. People in these areas are friendly					
7. Working in these areas results in "isolation" from friend and family					
8. Working as medical doctor in hospitals in these areas is the most important contribution to health of population					
9. Medical school prepared me well to work in these areas					
10. Medical education inspires me to work in hospitals in these areas					
11. There are abundant amenities and entertainment in these areas					
12. Working in hospitals in these areas is most challenging					
13. Working in hospital in these areas provide opportunities for real-life problem solving					

4. Job preferences upon graduation

4.1 You are about to graduate, where are you intending to work? (Please choose one answer only)

- ❑1. Health service in public sector
- ❑2. Health service in private hospital
- ❑3. Public academia (further training, research or teaching)
- ❑4. Private academia (further training, research or teaching)
- ❑5. Non government organizations (NGOs)/ Disadvantaged community (rural, urban slum)
- ❑6. Outside my country
- ❑7. Other, specify.....

4.2 What three most important reasons made you intend to work there? Please rank “1” for the first important reason and “2” and “3” for the second and third reasons respectively

Reasons for intending to work in 4.1	Ranking
a. Make most money	
b. Get away from parents	
c. Live close to parents/ families	
d. Nice place to live	
e. Return to hometown	
f. Good career prospects	
g. Good welfare	
h. Opportunity for further training	
i. Work environment	
j. My own conception of social accountability	
k. Others, specify……………….	

4.3 Where do you see yourself working in the next 5 years?

❑1. Health service in public sector

❑2. Health service in private hospital

❑3. Public academia (further training, research or teaching)

❑4. Private academia (further training, research or teaching)

❑5. Non government organizations (NGOs)/ Disadvantaged community (rural, urban slum)

❑6. Outside country

❑7. Quit professional career

❑8. Other, specify.....

5. Competency self-assessment

Please score 1-5 from the least to the most confident

	← Least confident ----most confident →				
	**1**	**2**	**3**	**4**	**5**
1. Public health services					
2. Health administration					
3. Communication with community and professional peers					
4. Inter-professional collaboration					
5. Managing internal medicines patients					
6. Managing obstetric and gynecology patients					
7. Managing pediatric patients					
8. Managing surgical patients					
9. Managing general patients such as DM,					
10. Managing difficult labour and delivery					
11. Referring patients to upper level					
12. Overall clinical competency					
13. Overall public health competency					

6. School Facilities

How about facilities of your school, in your perception are there adequate for students? Please score 1-5 from non-existence to highly adequate

School Facilities	←Non-existence------Highly adequate→				
	1	2	3	4	5
1. Building					
2. Library services					
a) Library and information support					
b) Inter-library services					
3. Teaching facilities					
a) Classrooms					
b) Teaching labs (displays)					
c) Interactive Laboratories e.g. Basic science simulation/ anatomy simulation etc.					
4. IT facilities and services					
a) Computers per student					
b) Internet service					
c) Conference call technology					
d) Video conference technology					
e) Telemedicine/ Tele-radiology link					
5. Training on use of IT					
a) Library search training course					
b) Computer skill lab					
c) Other IT training course specify					
6. Field sites					
7. Learning materials					
8. Accommodation for students , transport,					
10. Student health services					
11. Internal transportation for students					
12. Amenities e.g. cafeteria, convenient					
13. Sport and recreational facilities					

7. Students financial issues

7.1 How much did you pay for your education fees / tuition fees for the entire course of medical education? ……………………… National Currency Unit

7.2 How did you pay?

- ❑ 1. Lump sum
- ❑ 2. Installment
- ❑ 3. Other, specify.....

7.3 What was the source of the payment? (more than one answer applies) [Please modify answers according to country contexts]

- ❑ a. Government
- ❑ b. Scholarship
- ❑ c. Parents
- ❑ d. Family members or friends/relatives
- ❑ e. Loan from bank
- ❑ f. Educational loan in a special project/condition
- ❑ g. Trust-fund
- ❑ h. Part-time job
- ❑ i. Sold the farm/house/assets
- ❑ j. Other, specify.....

7.4 Did you suffer financial hardship in attending school?

- ❑ 1. No
- ❑ 2. Yes

7.5 Did you consider quitting/dropping out because of difficulty financing your education?

- ❑ 1. No
- ❑ 2. Yes
